# Stimulus-parity synaesthesia versus stimulus-dichotomy synaesthesia: Odd, even or something else?

**DOI:** 10.1068/i0702sas

**Published:** 2015-03-02

**Authors:** Rebekah C. White, Anna Plassart

**Affiliations:** Department of Experimental Psychology, University of Oxford, South Parks Road, Christ Church, Oxford OX1 1DP, UK; e-mail: Rebekah.White@psy.ox.ac.uk; Faculty of History, University of Oxford, George Street, Christ Church, Oxford, OX1 1DP, UK; e-mail: Anna.Plassart@chch.ox.ac.uk

**Keywords:** concurrent, inducer, parity, personification, synaesthesia

## Abstract

In stimulus-parity synaesthesia, a range of stimuli—for example, letters, numbers, weekdays, months, and colours (the inducers)—elicit an automatic feeling of oddness or evenness (the concurrent). This phenomenon was first described by Théodore Flournoy in 1893, and has only recently been “rediscovered.” Here, we describe an individual who experiences a comparable phenomenon, but uses the labels negative and positive rather than odd and even. Stimulus-parity synaesthesia may be broader than first supposed, and it is important that assessments are sensitive to this breadth.

*Is the word “November*” *a male or female? Is it odd or even?* These questions, rather than the word November, will likely strike many readers as odd. And yet, for individuals with certain subtypes of synaesthesia, the questions elicit an automatic response. In ordinal linguistic personification (OLP: Simner & Holenstein, 2007), also referred to as sequence–personality synaesthesia (Simner, Gätner, & Taylor, 2011), members of ordinal linguistic sequences are personified; for example, they may be ascribed gender, appearance, and personality. Thus, the word November may elicit the image of a handsome, extroverted man. In stimulus–parity synaesthesia (White & Plassart, in press), stimuli elicit an automatic feeling of oddness or evenness. Thus, the word November may feel “very odd” or “slightly even.”

The term synaesthesia—from *syn-* (joining) and *-aesthesia* (sensation)—stresses sensation, but the phenomenon is remarkably broad, and comprises atypical merging of cognitive and sensory constructs (Simner, 2012). The triggering stimulus is called the inducer and the resulting experience is called the concurrent (Grossenbacher & Lovelace, 2001). There are at least 63 reported subtypes of synaesthesia (Day, 2014), with different conceptual and perceptual inducer-concurrent pairings. The most widely-investigated subtype is grapheme–colour synaesthesia, in which numbers and letters are associated with an experience of colour. Thus, a grapheme–colour synaesthete may perceive the letter N as orange and, due to the letter-to-word transference effect (Calkins, 1893; Simner & Holenstein, 2007; Simner & Hubbard, 2006), she may also perceive the word November as being orange. Although at first glance, grapheme–colour synaesthesia may appear to be a purely perceptual phenomenon— *seeing* graphemes elicits an experience of *seeing* colours—for the vast majority of synaesthetes, it is the conceptual notion or categorisation of the grapheme that elicits the colour experience (Simner, 2007), rather than low-level features of the grapheme (see also Ward, Li, Salih, & Sagiv, 2007). Indeed, Simner (2012) notes that the “overwhelming majority of synaesthesiae appear to be triggered by the high-order cognitive constructs involved in language comprehension and production” (p. 3, see also Simner, 2007). In OLP and stimulus-parity synaesthesia, high-order cognitive constructs serve as both the inducing stimulus and the concurrent experience. Some researchers have questioned whether the term synaesthesia should be reserved for phenomena involving perceptual concurrents.^1^ However, the less prototypical subtypes—such as those pairing conceptual inducers and conceptual concurrents—exhibit the hallmarks of synaesthesia. OLP associations have been shown to (1) co-occur with other subtypes, (2) be highly consistent, (3) have the characteristic of letter-to-word transference and (4) be automatically generated (Simner & Holenstein, 2007). And likewise, stimulus–parity associations have been shown to (1) co-occur with other subtypes, (2) be highly consistent and (3) elicit a subjective impression of automaticity (White & Plassart, in press).

Whereas OLP is a relatively well-known phenomenon, which has been the focus of historical (e.g. Calkins, 1893; Flournoy, 1893) and contemporary research (e.g. Amin et al., 2011; Simner & Holenstein, 2007; Simner et al., 2011), stimulus–parity synaesthesia is virtually unknown. The phenomenon was described by Flournoy in his 1893 book *Des phénomènes de synopsie* [“Of synoptic phenomena”]. Flournoy observed that many individuals attribute oddness and evenness to non-numerical stimuli, and he suggested that the attribution of oddness and evenness to weekdays may occur “even more frequently” (p. 222) than the attribution of gender to weekdays (as in OLP). Thus, it is surprising that 120 years passed without further discussion or observation of the odd–even phenomenon. We recently identified two individuals (R and M) who reported that a range of stimuli—for example, letters, numbers, weekdays, months, colours—elicited a feeling of oddness or evenness, and we offered the term stimulus–parity synaesthesia to describe this “forgotten” subtype of synaesthesia (White & Plassart, accepted manuscript).

Subsequently, we have identified another individual, W (a 63-year-old right-handed female), who experiences a near-identical phenomenon. Many stimuli—for example, letters, numbers, weekdays, months, seasons, city names—elicit an automatic feeling of belonging to one of two conceptual categories, and this occurs when W reads, hears or thinks about the stimulus; thus, it is the high-level conceptual stimulus that serves as the inducer. Crucially however, the two categories are “negative and positive,” rather than odd and even. When she uses the labels negative and positive, W is not referring to things as being bad or good. Some of her favourite seasons and places elicit the description negative. W explains that negative things elicit an automatic feeling of being “soft, subtle, gentle, uncluttered, delicate, transient, and with fuzzy edges” whereas positive things elicit an automatic feeling of being “strong, defined, inflexible and structured”. We assessed W on two occasions, separated by 2.5 months. At each testing session, we presented W with a list of 67 stimuli (letters = 26, numbers = 10, weekdays = 7, months = 12, seasons = 4, city names = 8), and asked her to indicate whether each stimulus was negative or positive. Responses were highly consistent at the two time points, with identical responses for 66/67 stimuli (98.51%).

W's associations have the hallmarks of synaesthesia. Individuals with one form of synaesthesia are likely to have another (Simner, 2012), and W also experiences OLP. Synaesthetic associations tend to be consistent across time, and W demonstrates highly consistent negative–positive associations. Concurrent experiences in synaesthesia are perceived as involuntary, and W reports that stimuli elicit an immediate feeling of being negative or positive. In addition, early onset is characteristic of synaesthesia, and W reports having experienced stimuli in this way for as long as she can remember.

Do W's negative–positive associations constitute a *new* subtype of synaesthesia? Although this is possible, a more parsimonious explanation is that the odd–even phenomenon that Flournoy (1893) first described, and that we have recently termed stimulus–parity synaesthesia (White & Plassart, in press), may be broader than first supposed. Individuals with this subtype of synaesthesia may ascribe all manner of dichotomous labels to eliciting stimuli: the labels that we have uncovered—odd and even, negative and positive—may only scratch the surface.

## Footnotes

1 For recent discussions about the breadth of phenomena that may constitute synaesthesia, and how these phenomena relate to normal crossmodal perception in non-synaesthetic individuals, see: Cohen Kadosh and Terhune (2012); Deroy and Spence (2013); Rothen and Meier (2013); and Simner (2012).
